# Quantum Dynamics of Rotational Transitions in CN (*X* ^2^Σ^+^) by H^+^ Collisions

**DOI:** 10.3389/fchem.2021.790416

**Published:** 2021-11-25

**Authors:** Bhargava Anusuri, T. J. Dhilip Kumar, Sanjay Kumar

**Affiliations:** ^1^ Department of Chemistry, Indian Institute of Technology Madras, Chennai, India; ^2^ Department of Chemistry, Indian Institute of Technology Ropar, Rupnagar, India

**Keywords:** Proton-CN, collisions, quantum calculations, rotational excitations, cross sections, interstellar medium

## Abstract

Collisional cross-sections of inelastic rotational excitations of CN in its ground electronic state (X^2^Σ^+^) by H^+^ scattering are studied by the exact quantum mechanical close-coupling (CC) method at very low collision energies (0–600 cm^−1^) relevant to interstellar atmospheres. *Ab initio* rigid rotor potential energy surface computed at MRCI/*cc*-*p*VTZ level of accuracy has been employed. Rate coefficients for the rotational excitations have also been calculated. The obtained results are compared with previous theoretical calculations and analyzed whether proton collisions could be significant sources for rotationally excited CN as a possible source for cosmic microwave background of about 3 K from the interstellar media.

## Introduction

As early as 1965, cosmic microwave background radiation (CMBR) measurements were carried out, and an analysis of radio wave intensities revealed that there exists approximately isotropic CMBR corresponding to black body radiation of about 3 K (Penzias and Wilson, 1965; Dicke et al., 1965; Stokes et al., 1967)^1^. These radiations are considered as remnant radiation occurring due to falling out of the big-bang fireball. The near isotropic nature of CMBR also suggested that the Universe is full of 3 K CMBR (Dicke et al., 1965).

Subsequently, the rotational temperature of interstellar CN molecule was measured (Field and Hitchcock, 1966; Thaddeus and Clauser, 1966) in terms of the population ratio of the first rotationally excited state to the ground rotational state (n_1_/n_0_), which was found to be 0.55 ± 0.05. Using $\frac{n_{1}}{n_{0}}=\frac{\left(2j^{\prime }+1\right)}{\left(2j+1\right)}e^{-\frac{\Delta E_{0\to 1}}{k_{b}T}}=0.5$ , where *j* stands for the rotational quantum number, $\Delta E_{0\to 1}$ is the energy difference of the two rotational energy levels (2 B = 2 × 1.8997 cm^−1^, B: diatomic rotational constant of CN, Huber and Herzberg, 1979), *k*_*b*_ (= 0.695 cm^−1^ K^−1^) is the Boltzmann constant, one estimates the rotational temperature of interstellar CN molecule to be approximately 3 K.

The fact that the rotationally-excited (*j’* = 1) CN molecule could be one of the primary sources for the CMBR (of about 3 K) has led to exploring various mechanisms and pathways that lead CN molecules to rotationally excited states. The CN radical has a permanent dipole moment, and therefore one expects it would have a higher probability for rotational excitations with electrons, protons, and ions. It can exist in two regions: 1) neutral hydrogen clouds (H-I region) and 2) ionized-hydrogen clouds (H-II region). In the H-I region, the cloud temperature can increase by several thousand degrees by cloud-cloud collisions, finally decreasing via infrared emissions. Here, the atomic hydrogen does not get ionized. However, minor constituents like Li, Mg, C, Si, Fe, etc., may get ionized liberating electrons through photoionization (Nishimura, 1968). Typically, the number density of these ions is approximated to be *N*_ion_ = *N*_e_ = 1.6 × 10^−3^ cm^−3^. Therefore, the rate of collisions of the ions (with CN) is expected to be minimal compared to that of the electron because of the latter’s lighter (thousand times smaller) mass. Another energy source for the H-I cloud can be from the low-energy (in MeV range) component of the cosmic-ray protons. If the flux is appropriate, they can ionize the atomic hydrogen present as the principal constituent in the cloud. In such conditions, the cloud temperature is estimated to be around 100 K (Hayakawa et al., 1961)^7^ with *N*_*e*_ = *N*_p_ = 0.03 cm^−3^.

In the H-II region, the gas exists in a completely ionized form. It gets heated up from lights emitted from stars up to a typical temperature of 10^4^ K. The gas density varies, resulting in high and low-density regions. An estimate of the low density could be around *N*_*e*_ = *N*_p_ < 0.1 cm^−3^.

Since the density of H^+^ is estimated to be an order of magnitude higher in the H-II region, it is appropriate to study the collisional excitation rates with CN molecules. Considering the temperature range of 100–1000 K of the gas in the H-II region, the collision energy (in the center of mass frame, E_c.m_) corresponds to 0.01 and 1.0 eV, respectively. Takayanagi and Itikawa (1968) carried out early theoretical calculations of rotational excitation of CN molecule by electron collisions using the close-coupling and the Born-approximation methods at E_c.m_ = 0.01, 0.1, and 1.0 eV. The interaction potential has been modeled in terms of the dipole, polarization, and short-range electrostatic interactions. Excitations by protons were studied in the classical framework using the trajectory impact parameter method. Later, Jamieson et al. (1975) studied the rotational excitations of CN molecule by proton collisions by impact parameter method using a modified interaction potential used earlier for the *e* ^−^ + CN system (Crawford et al., 1969; Allison and Dalgarno, 1971). This interaction potential was based on dipole and polarization interactions. They obtained approximate solutions for rotational excitations based on Born and the “exponential” approximations, and the obtained integral cross sections were compared with those obtained from the close-coupling method. The calculations were carried out for E_c.m_ = 0.04—500 eV. Since long-range interactions are present in *e* ^−^/H^+^ interactions with CN molecule leading to significant inelastic effects, it was desirable to test the validity of decoupling approximations of angular momenta to carry out efficient and reliable calculations. Subsequently, DePristo and Alexander (DePristo and Alexander, 1976) investigated decoupling approximations of angular momenta, namely decoupled *l*—dominant (DLD) method (DePristo and Alexander, 1975) using the model interaction potential. Various theoretical aspects were discussed, and cross-sections for rotational excitations were calculated at E_c.m_ = 0.04, 0.1 and 1.0 eV with dipole moments of CN, *μ* = 1.1 D and 1.45 D.

## Potential Energy Surface

The bound HCN^+^ ion has a collinear geometry. There are two energetically low-lying electronic states in the collinear geometry: 1^2^Σ^+^ and 1^2^Π. Asymptotically, The former correlates to the inelastic channel, H^+^ + CN(X^2^Σ^+^), and the latter to charge transfer channel, H (^2^S) + CN^+^ (^2^Σ^+^). The computed energy for the asymptotic charge transfer channel is 0.278 eV higher than that of the inelastic channel. Around the equilibrium geometry and the Franck-Condon region, the 1^2^Π state is lower in energy, and the computed energy difference between the 1^2^Π and the 1^2^∑ states is 0.979 eV (Anusuri and Kumar, 2016). For the off-collinear geometry, the 1^2^Π state splits into the ^*2*^ *A′* and ^*2*^ *A″* states. The ^2^Σ^+^ state correlates to the ^*2*^ *A’* state. The nondegenerate states’ potential energy surfaces (PES) interact in the vicinity of collinear geometry with off-collinear distortions. Thus, the bound HCN^+^ constitutes a Renner-Teller system (see, KÖppel et al., 1979; Perić et al., 1983; and references therein), exhibiting strong vibronic interactions between the ground ^2^Π and the first excited ^2^Σ^+^states. The existence of Renner-Teller coupling complicates the assignment of vibrational progressions. For a brief account of the vibrational progression studies, see reference (Anusuri and Kumar, 2016) and the references therein.

**FIGURE 1:**
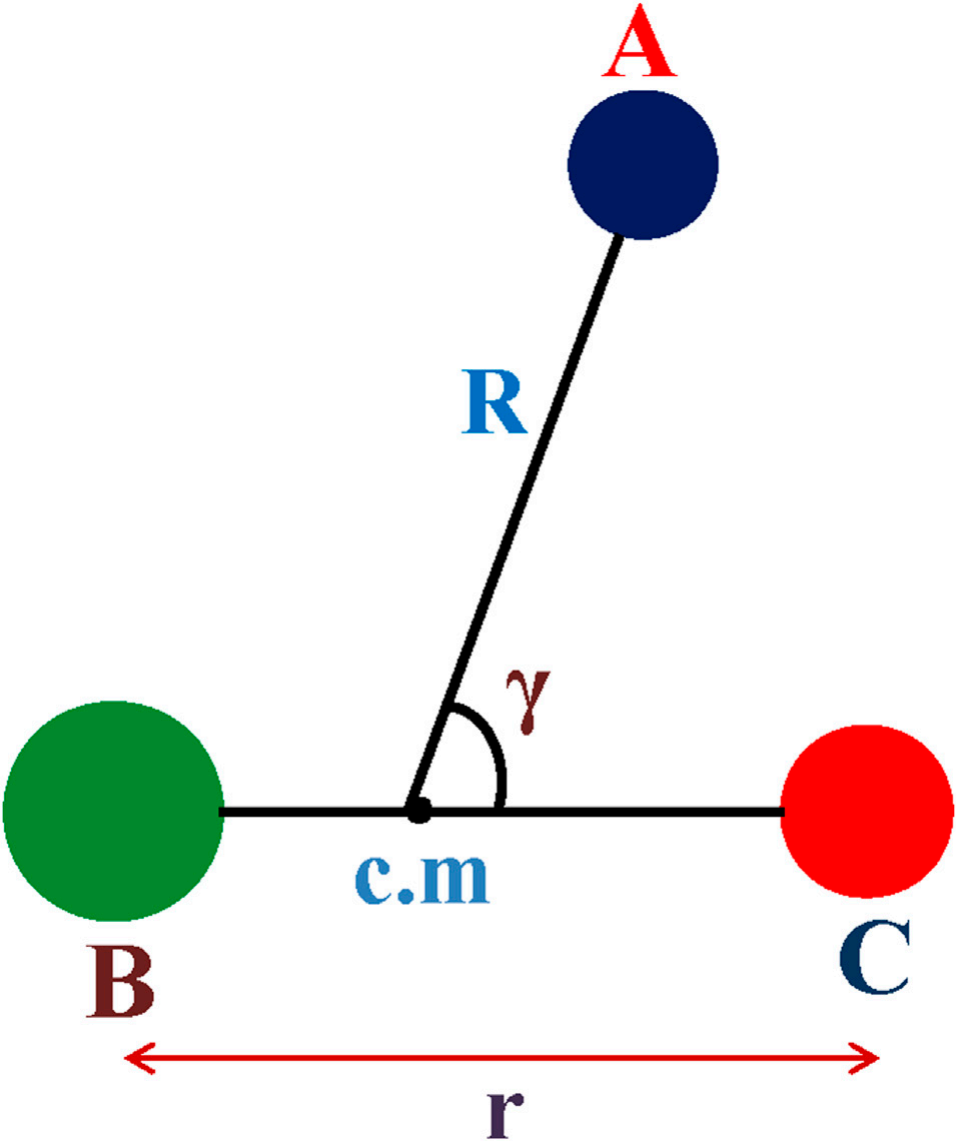
Jacobi scattering coordinates.

For the scattering studies involving the inelastic channel, H^+^ + CN(X^2^Σ^+^), it is crucial to examine the (nonadiabatic) involvement of the 1^2^Π state, which correlates to the endoergic charge transfer channel, H (^2^S) + CN^+^ (^3^Π). Since only the inelastic rotational excitations of the CN molecules upon collisions of H^+^ are studied, the potential energy surface is generated in the Jacobi scattering coordinates (Figure 1): **r** is the interatomic distance of the diatom BC (CN), **R** is the distance of H^+^ from the center of mass of BC (CN), and γ = cos^−1^ (**R**.**r**) is the angle between **R** and **r**. H^+^ approaching C-end is considered as γ = 0° and N-end as γ = 180°. Our earlier study (Anusuri and Kumar, 2016) found that the potential energy curve of 1^2^Π state crosses with that of the 1^2^Σ^+^ state around R = 5*a*_o_ in the collinear geometry. For off-collinear geometry, the energy order is $1^{2}A^{\prime }<1^{2}A^{\prime \prime }<2^{2}A^{\prime }$ . The $A^{\prime }$ states correlate to the inelastic channel while the $A^{\prime \prime }$ correlates to the charge transfer channel. The two $A^{\prime }$ states are reasonably well separated energetically. There is no radial coupling between the $1A^{\prime }\;$ and $1A^{\prime \prime }$ states. The radial coupling between the $1A^{\prime }\;$ and $2A^{\prime }$ states are significantly small for $r=r_{eq};\;$ It shows up little strength in the repulsive regions of the PESs and for stretched values of *r*. However, the nonadiabatic interactions arising through the spin-orbit coupling for the collinear geometries and the radial couplings for close-collinear geometries may not be ignored. Considering that the scattering processes of the present study do not involve any charge transfer outcome and that we are interested only in the low rotational excitations, we believe that the nonadiabatic interactions of the higher states would be significantly less, and scattering the rigid-rotor surface of the 1^2^Σ^+^/ $1A^{\prime }$ states would largely capture the collision dynamics. Therefore, we describe below the details of the rigid rotor PES computations.

Calculations were carried out in the *C*_2υ_ symmetry for collinear geometries and the *C*_s_ symmetry for off-collinear geometries. The ground state RR surface was computed for the 1^2^Σ^+^/1^2^ *A′* symmetry for the collinear/off-collinear approaches. Computations were done at internally contracted multi-reference configuration interaction (Knowles and Werner, 1988; Werner and Knowles, 1988; Knowles and Werner, 1992) level of accuracy with Dunning’s (Dunning, 1989) *cc*-*p*VTZ basis set using MOLPRO 2010.1 (Werner et al., 2010) suite of programs. A total of 780 geometries were computed with *r* fixed at *r*_*eq*_ = 2.23 *a*_*o*_: γ = 0°–180° (15°); R = 0.8–1.8 (0.2), 1.9–4.0 (0.1), 4.2–7.0 (0.2), 7.5–10.0 (0.5), 11.0–15.0 (1.0). R is in atomic units, and the numbers in the parentheses indicate the step size in the interval.

The computed basis set superposition error (BSSE) was small and varied systematically in the range 0.04–0.01 eV for R 3.0 *a*_*o*_ to 5.0 *a*_*o*_, and falls off rapidly and becomes negligible (less than 10^−4^ eV) for R > 5.0 *a*_*o*_. R = 3.0 *a*_*o*_ approximately signifies the bottom of the interaction well. We believe that the BSSE correction will still have a very small correction on the computed cross sections and rates, and therefore, we did not include them. The effect of the augmented basis set was also very small as interaction potential values varied on the second decimal places in eV. We believe that the potential energy surface generated using the cc-*p*VTZ basis set is sufficiently accurate for the scattering calculations.

**FIGURE 2:**
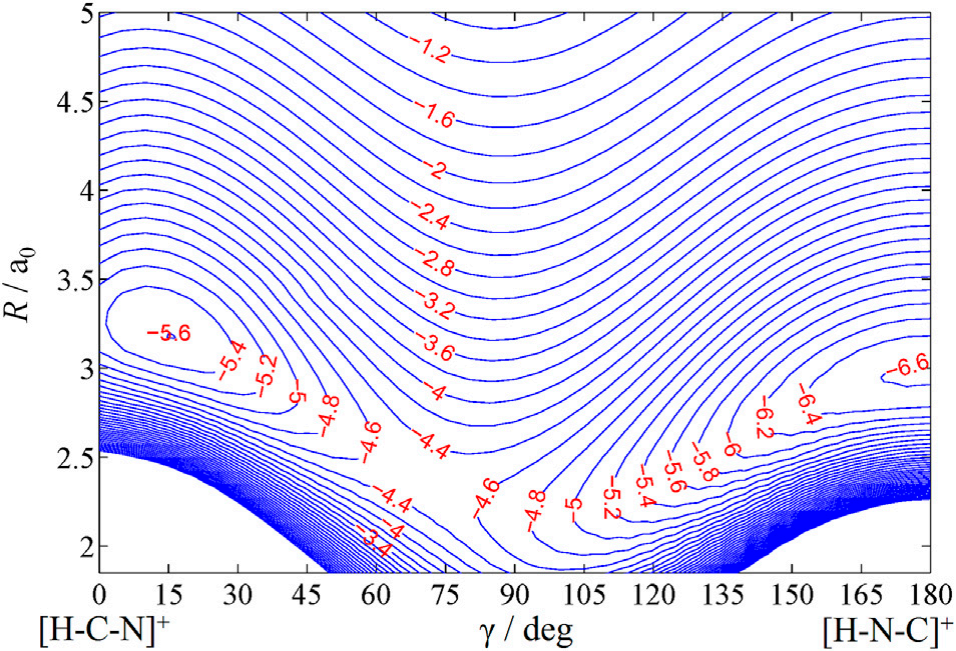
Contour plot of rigid-rotor PES of HCN^+^ (1^2^Σ^+^/ $1^{2}A^{\prime }$ ) as a function of *R* and γ with *r* fixed at *r*_eq_ = 2.23 *a*_*0*_. The spacing between the contours is 0.2 eV.

The contour plot of the rigid-rotor surface is shown in Figure 2. To study the dynamics, we need the long-range asymptotic interaction potential up to large *R* values where the interaction potential dies down. For large values of *R*, the interaction potential is generally expanded in multipolar terms. Here, the charge on H^+^ interacts with the dipole, quadrupole, and polarizability components of CN, and the asymptotic interaction potential $V_{as}\left(R\right)$ is given as:

$$V_{as}\left(R\right)∼\frac{\mu }{R^{2}}P_{1}\left(\cos\gamma \right)+\frac{Q}{R^{3}}P_{2}\left(\cos\gamma \right)-\frac{\alpha_{0}}{2R^{4}}-\frac{\alpha_{2}}{2R^{4}}P_{2}\left(\cos\gamma \right)+O\left(P_{3}\right)$$ (1)

where *μ* (−1.362 D) is the dipole moment, *Q* (0.368 $ea_{0}^{2}$ ) is the quadrupole moment, *α*_*0*_ (17.92 $a_{0}^{3}$ ) and *α*_*2*_ (10.58 $a_{0}^{3}$ ) are the polarizability components of CN(X^2^Σ^+^) at *r* = *r*_*eq*_. *P*’s are the Legendre polynomials. The mentioned values were obtained by the *ab initio* calculations using the same basis set and the computational methodology.

### Multipolar Expansion

The rigid-rotor interaction potential has been fitted to the following analytic expression:

$$V\left(R,r=r_{eq},\gamma \right)=\underset{\lambda }{\sum }V_{\lambda }\left(R\right)P_{\lambda }\left(\cos\gamma \right)$$ (2)

where *V*_*λ*_’s are expansion coefficients with λ varying from 0 to 12 (number of γ values) and *P*_*λ*_’s are Legendre polynomials.

**FIGURE 3:**
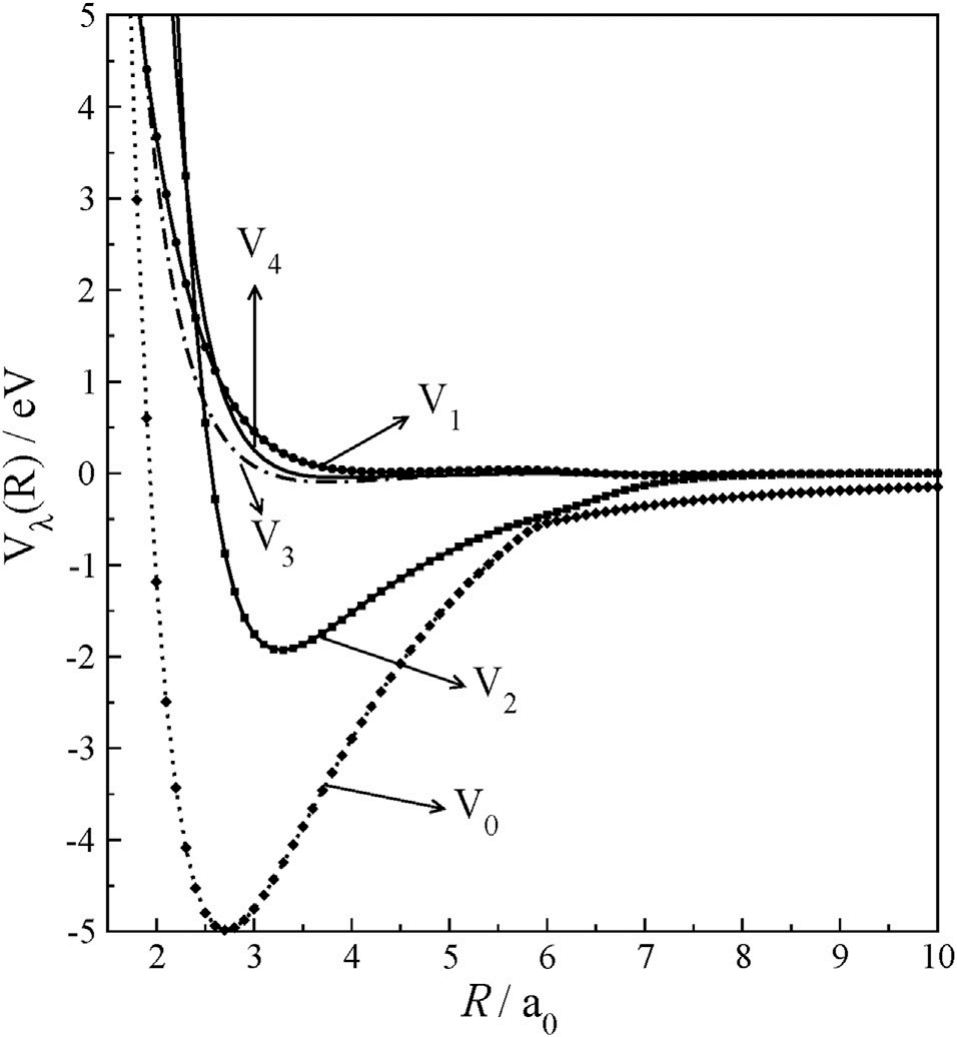
Radial multipolar expansion coefficients of HCN^+^ rigid-rotor potential as a function of *R* at *r* = *r*_eq_.

*V*_*λ*_ were numerically obtained at the computed grid points using the Legendre polynomials’ orthogonality. Once their dependence on grid points of *R* is known, each *V*_*λ*_ was fitted with a cubic spline fit. The interaction anisotropy can be examined in terms of *V*_*λ*_’s plotted as a function of *R* and shown in Figure 3. *V*_0,_ which gives spherically averaged interaction potential, exhibits a deep well and extends its strength beyond *R* = 10.0 *a*_0_ in long-range interactions given in charge-multipole interactions. Interestingly, *V*_1_, *V*_3,_ and *V*_4_ components are very small throughout *R,* and they are repulsive and show their strength only at closer approaches. Next to *V*_0_, *V*_2_ only shows an interaction well. Therefore, it suggests that rotational excitations at low collision energies will be governed by only a few low *V*_*λ*_’s.

## Scattering Calculations

The molecular energy levels in CN (*X* ^2^Σ^+^) are described by Hund’s case 2) limit. The theoretical framework for the scattering of a molecule in ^2^Σ^+^ electronic state was given by Alexander (Alexander, 1982). The rotational fine structure levels in the CN molecule here are labeled by the quantum numbers *j* and *J* *. j* is the rotational angular momentum, whereas *J* is the total angular momentum*. J* is given by *J = j + s* where *s* is the electronic spin angular momentum. For CN molecule in ^2^Σ^+^ electronic state, the *J* levels will be *J* = *j + ½* (labeled as *e*) and *J = j—½* (labeled as *f*).

Full close-coupling calculations have been carried in very low collision energy range of 0–600 cm^−1^ for rotational excitations from *j = 0* to *j' =* one to four levels. Time-independent coupled scattering equations (Arthurs and Dalgarno, 1960) have been solved to compute cross-sections implemented in the HIBRIDON package (Alexander et al., 2011). The close-coupled radial equations were numerically integrated using the log derivative propagator (Manolopoulos, 1986). The following input parameters are taken in the calculation: rotational constant, *B*_*e*_ = 1.89102 cm^−1^, *D*_0_ (centrifugal distortion constant) = 6.4 × 10^−6^ cm^−1^, spin-splitting constant γ_0_ = 7.2549 × 10^−3^ cm^−1^ (Huber and Herzberg, 1979) and reduced mass of the system, *μ* = 0.970,404 a.u. with values of *R*_min_ and *R*_max_ as 1.4 and 200 *a*_0_, respectively with Δ*R* = 0.1 *a*_0_. The value of CN bond length is fixed at 2.23 *a*_0_ (rigid-rotor approximation). The main focus of the present study is to have the first meaningful yet reliable estimates of the cross sections for rotational excitation for *j* = 0 - *j′* = 1, using the full quantum calculations in vibrational manifold v = 0. Therefore, rigid-rotor calculations would capture the collision dynamics since there would hardly be any centrifugal distortions in the CN bond the small rotational excitation(s). The cross sections are computed for energies up to 600 cm^−1^. At least three energetically closed rotational channels were included in the calculations at a particular E_c.m_ to ensure numerical convergence of cross section within the acceptable limit (up to third decimal place). Maximum value of rotational quantum number taken is *j*_max_ = 18 at E_c.m_ = 600 cm^−1^. Also, the convergence of cross-sections is achieved through a sufficient number of partial waves; for instance, at E_c.m_ = 500 cm^−1^, *l* (total angular momentum) is kept at 580. The CC calculations have been performed from an energy value corresponding to the opening of the lowest inelastic channel to a total energy of 600 cm^−1^.

### Integral Cross-Sections

**FIGURE 4:**
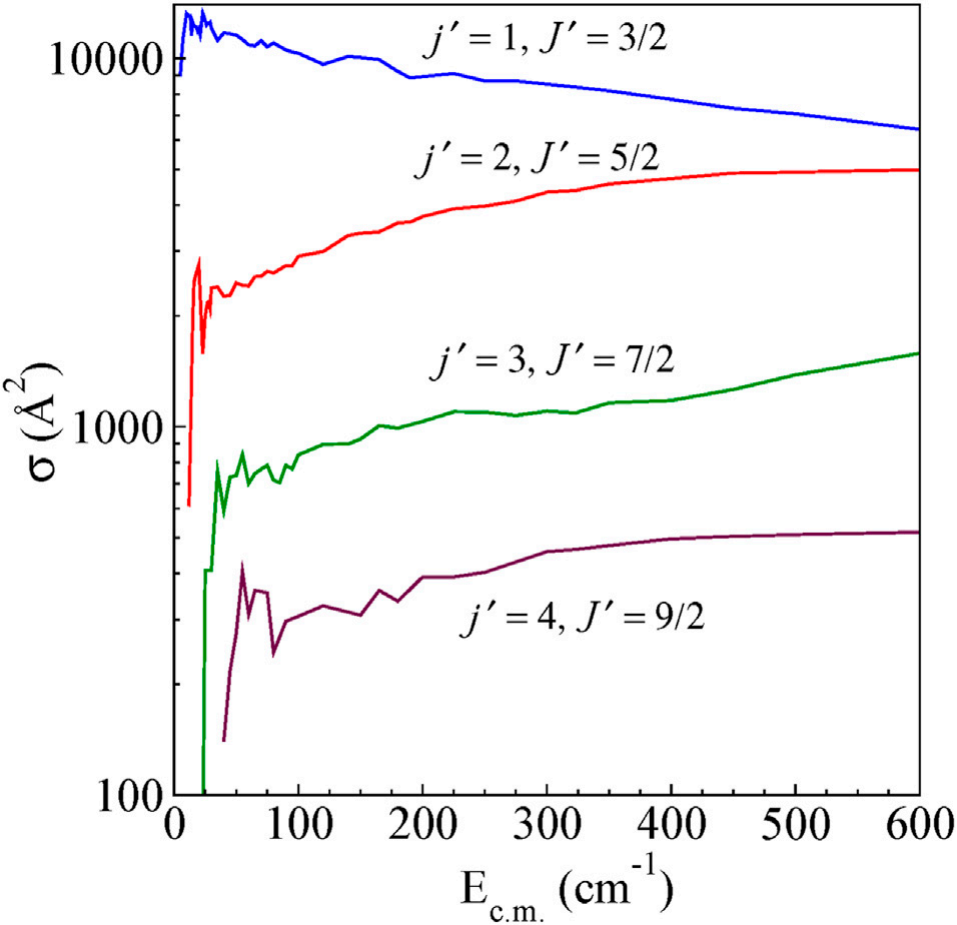
State-to-state integral cross sections for rotational excitations for *ΔJ = Δj* transitions from the initial level *j =* 0 and *J =* ½ for H^*+*^ *+* CN (*j =* 0*, J =* ½) *→* H^*+*^ *+* CN (*j′, J′*)*.*

**FIGURE 5:**
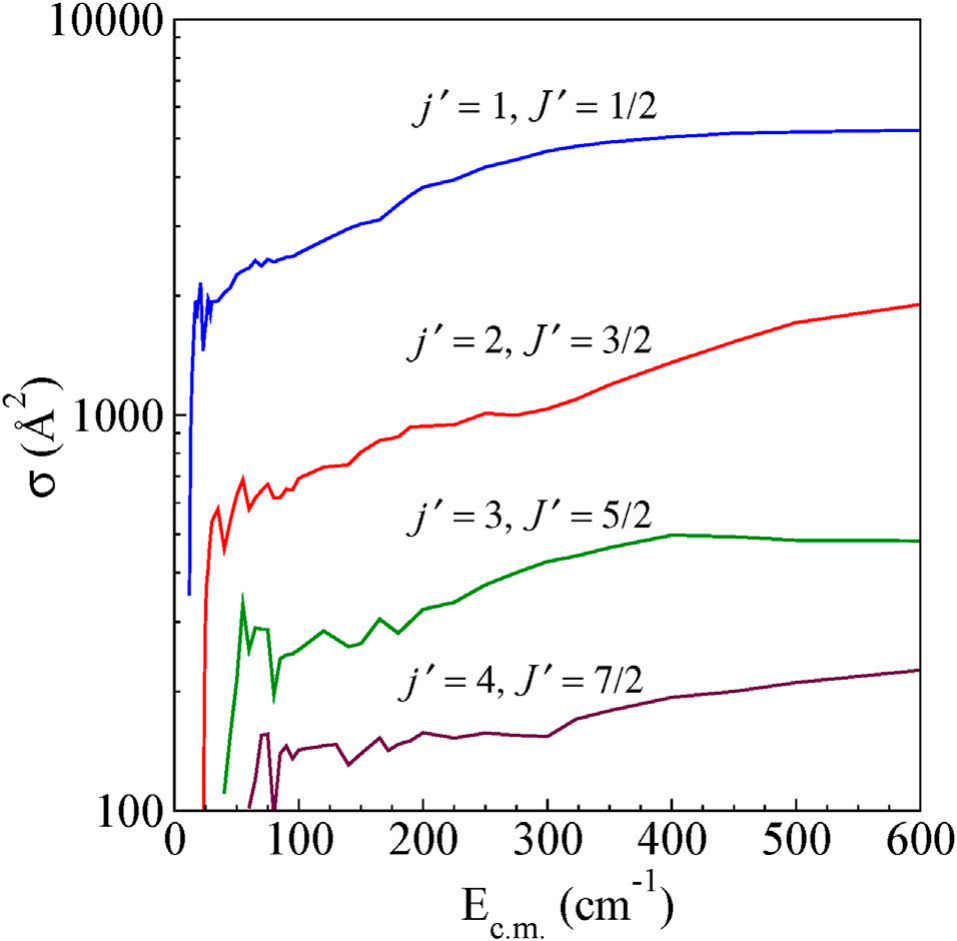
State-to-state integral cross sections for rotational excitations for *ΔJ ≠ Δj* transitions from the initial level *j =* 0 and *J =* ½ for H^*+*^ *+* CN (*j =* 0*, J =* ½) *→* H^*+*^ *+* CN (*j′,J′*)*.*

The integral cross-sections for rotationally inelastic excitations obtained from the full close-coupling calculations in the energy range (5–600 cm^−1^) are shown as a function of E_c.m_ for excitations from the ground (*j* = 0, *J* = 1/2) rotational level to higher rotational levels for ΔJ = Δ*j* transitions and Δ*J* ≠ Δ*j* transitions in Figure 4 and Figure 5, respectively. The rotational excitations are plotted for *j* = 0 → *j'* = one to four levels. In the case of *j* = 0 to *j'* = 1 transition, the cross sections oscillate at low energies for both Δ*J* = Δ*j* as well as for Δ*J* ≠ Δ*j*. The cross section for Δ*J* = Δ*j* from the energetic threshold rises to a maximum and shows a large decreasing plateau with increasing collision energy. For Δ*J* ≠ Δ*j* the cross sections show a monotonic increasing behavior with an increase in E_c.m_. Near the threshold, all the cross sections show oscillatory behavior presumably due to the formation of scattering resonances. We have also observed a propensity for Δ*J* = Δ*j* transitions compared to that of Δ*J* ≠ Δ*j* transitions, which is a common feature in the scattering of molecules in ^2S+1^Σ electronic state (Alexander et al., 1986). This propensity is reported in He-CN scattering also (Lique et al., 2010).

The total state-to-state (*j*´) cross sections were obtained by summing the cross sections for the Δ*J* = Δ*j* and Δ*J* ≠ Δ*j* transitions. The computed integral cross sections for 0 → 1 and 0 → 2 are compared in Table 1 and earlier theoretical estimates. The earlier theoretical values were reported in units of cm^2^. Therefore, we also report our values in the same units for better comparison and convenience.

**TABLE 1:** Comparison of the present CC cross section results with literature data.

Collision energy E_c.m_ (eV)	Present study		Jamieson et al., 1975 (CC)		DePristo and Alexander, (1976)	
	σ_0→1_ (10^−12^ cm^2^)	σ_0→2_ (10^−14^ cm^2^)	σ_0→1_ (10^−12^ cm^2^)	σ_0→2_ (10^−14^ cm^2^)	σ_0→1_ (10^−12^ cm^2^)	σ_0→2_ (10^−14^ cm^2^)
0.04	1.31	54.7	0.48	0.26	0.79	4.4
0.06	1.23	65.7	1.22	2.02	—	—
0.08	2.28	138.2	1.89	7.04	—	—

The cross sections are compared at three collision energies, E_c.m_ = 0.04, 0.06 and 0.08 eV. The 0 → 1 cross sections compare reasonably well with the previous calculations for these energies. The cross sections for 0 → 2 excitations are approximately two orders of magnitude less than that of 0 → 1 excitations. The present calculations yield consistently higher magnitudes for 0 → 2 excitations than those obtained in earlier calculations. The 0 → 1 cross sections are mostly governed by long-range effects (Harrison et al., 2012). Since the long-range interaction potential description is similar for the earlier and the present studies, there is an overall good agreement. But the direct 0 → 2 transitions are governed by the V_2_ multipolar term, the next strongest term after the V_0_ term in the interaction potential expansion.

### Rate Coefficients

**FIGURE 6:**
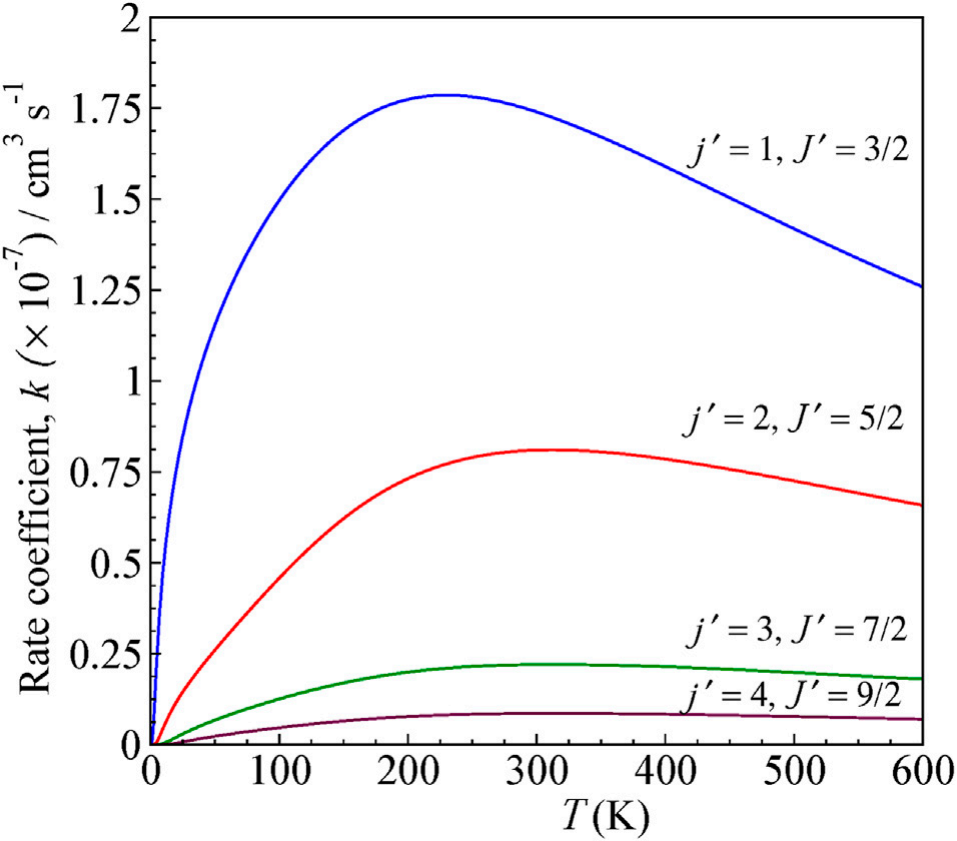
State-to-state rate as a function of temperature for 0–600 K computed for Δ*J* = Δ*j* transitions from the initial level *j =* 0 and *J =* ½ for H^*+*^ *+* CN (*j =* 0*, J =* ½) *→* H^*+*^ *+* CN (*j', J′*)*.*

**FIGURE 7:**
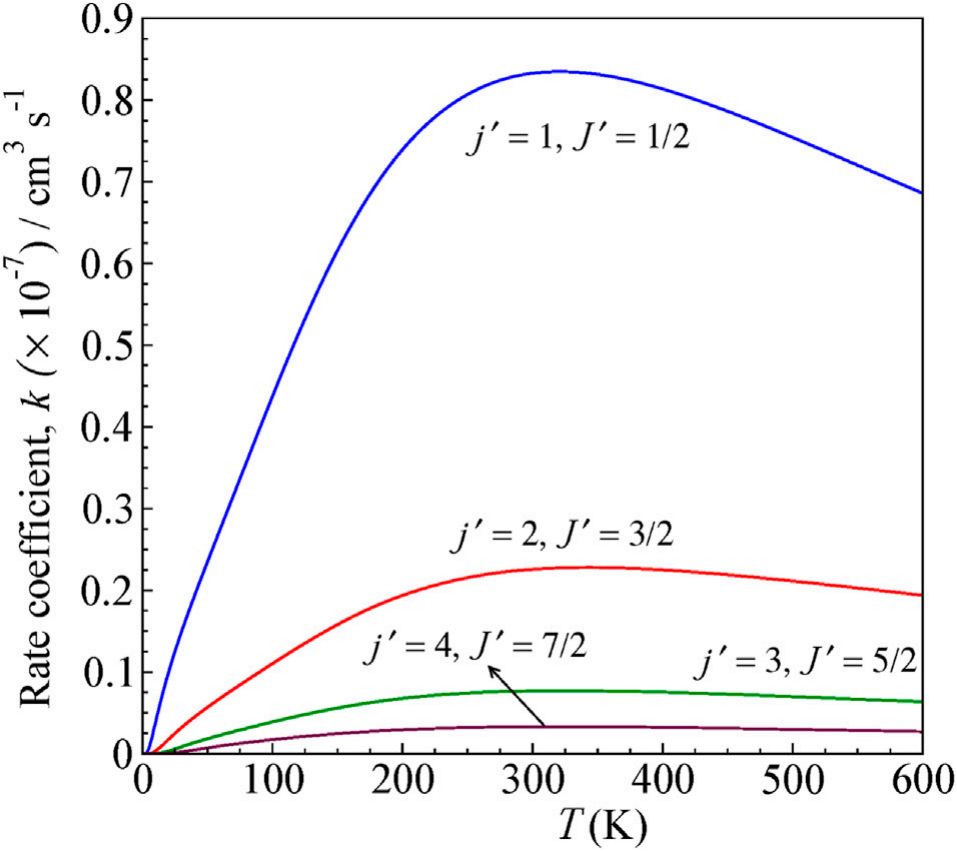
State-to-state rate as a function of temperature for 0–600 K computed for Δ*J* ≠ Δ*j* transitions from the initial level *j =* 0 and *J =* ½ for H^*+*^ *+* CN (*j =* 0*, J =* ½) *→* H^*+*^ *+* CN (*j',J′*)*.*

The computed rotational cross-sections are used to calculate rate-coefficient as a function of temperature:

$$k_{N,j\to N^{\prime }j^{\prime }}\left(T\right)=\sqrt{\frac{8k_{B}T}{\pi \mu }}{\left(\frac{1}{k_{B}T}\right)}^{2}\overset{\infty }{\underset{0}{\int }}\sigma \left(E\right)Ee^{\left(-E/k_{B}T\right)}dE$$ (3)

where *k*_*B*_ is the Boltzmann constant, *μ* is the reduced mass of the system, and E = E_c.m_. The state-to-state rate coefficients for rotational transitions over a range of temperatures computed for both Δ*J* = Δ*j* and Δ*J* ≠ Δ*j*, up to *T* = 600 K, are shown in Figure 6 and Figure 7, respectively.

The rates are higher for *j = 0 → j′ = 1* transition, similar to cross sections, and decreases for other higher excitations. The rate coefficients are calculated by averaging the obtained cross section over a Boltzmann distribution of kinetic energy.

The rate coefficient for *j = 0* to *j′ = 1* excitation in CN by proton scattering was estimated to be about the order of 10^−7^ cm^3^ s^−1^ by Jamieson et al., 1975 at 80 K, which is the temperature of interstellar clouds. It is lower by a factor of ten compared to that obtained from the electron scattering calculations with CN. In our present calculation, we obtained a rate coefficient of 1.74 × 10^−7^ cm^3^ s^−1^.

## Summary and Conclusions

*Ab initio* rigid-rotor PES obtained at MRCI/*cc*-*p*VTZ level of theory has been employed to study the inelastic rotational excitations in CN by H^+^ scattering. The ground electronic state PES asymptotically correlates to H^+^ (^1^S) + CN (^2^Σ^+^) for H^+^ + CN system. The contour plot of [HCN]^+^ shows deeper interaction wells corresponding to two collinear configurations. The long-range interaction potential is obtained in charge–dipole, -quadrupole, and polarizability interactions. The potential anisotropy of the system has been analyzed in terms of radial multipolar expansion. The rotational transitions are studied on the PES for low collision energies between 0—600 cm^−1^ by solving close-coupled equations. The cross-sections are calculated for inelastic rotational transitions for *J* = 0 → *J'* = 1-4 one to four using close-coupling method.

The rate coefficients in the temperature range 0–600 K have been calculated. The rate coefficients for *j* = 0 → *j*’ = 1 transition in H^+^ scattering of CN is found to be of the order of 10^−7^ cm^3^ s^−1^. It is an order of magnitude less than that of electron scattering at the average temperature of interstellar clouds (∼80 K). In clouds of the H-II region where H^+^ density could be relatively larger (>> 0.1 cm^−3^) H^+^ collision could lead to 0 → 1 rotational excitation of CN, which could become comparable to that obtained in collision with electrons.

## Data Availability

The raw data supporting the conclusion of this article will be made available by the authors, without undue reservation.

## Footnotes

1 Dedicated to Professor Saurav Pal, Director, IISER Kolkata, India.
